# A study on the ultimate mechanical properties of middle-aged and elderly human aorta based on uniaxial tensile test

**DOI:** 10.3389/fbioe.2024.1357056

**Published:** 2024-03-21

**Authors:** Hongbing Chen, Minzhu Zhao, Yongguo Li, Qi Wang, Yu Xing, Cunhao Bian, Jianbo Li

**Affiliations:** ^1^ Department of Forensic Medicine, College of Basic Medicine, Chongqing Medical University, Chongqing, China; ^2^ Chongqing Engineering Research Center for Criminal Investigation Technology, Chongqing, China; ^3^ Chongqing Key Laboratory of Forensic Medicine, Chongqing, China

**Keywords:** human aorta, uniaxial tensile test, material properties, pathology, atherosclerosis

## Abstract

**Background:**

The mechanical properties of the aorta are particularly important in clinical medicine and forensic science, serving as basic data for further exploration of aortic disease or injury mechanisms.

**Objective:**

To study the influence of various factors (age, gender, test direction, anatomical location, and pathological characteristics) on the mechanical properties and thickness of the aorta.

**Methods:**

In this study, a total of 24 aortas (age range: 54–88 years old) were collected, one hundred and seventy-four dog-bone-shaped samples were made, and then the uniaxial tensile test was run, finally, pathological grouping was performed through histological staining.

**Results:**

Atherosclerotic plaques were mainly distributed near the openings of blood vessel branches. The distribution was most severe in the abdominal aorta, followed by the aortic arch. Aortic atherosclerosis was a more severe trend in the male group. In the comparison of thickness, there were no significant differences in age (over 50 years) and test direction, the average thickness of the aorta was greater in the male group than the female group and decreased progressively from the ascending aorta to the abdominal aorta. Comparing the mechanical parameters, various parameters are mainly negatively correlated with age, especially in the circumferential ascending aorta (ε_p_ “Y = −0.01402*X + 1.762, R^2^ = 0.6882”, ε_t_ “Y = −0.01062*X + 1.250, R^2^ = 0.6772”); the parameters of males in the healthy group were larger, while the parameters of females were larger in atherosclerosis group; the aorta has anisotropy, the parameters in the circumferential direction were greater than those in the axial direction; the parameters of the ascending aorta were the largest in the circumferential direction, the ultimate stress [σ_p_ “1.69 (1.08,2.32)”] and ultimate elastic modulus [E_2_“8.28 (6.67,10.25)”] of the abdominal aorta were significantly larger in the axial direction; In the circumferential direction, the stress [σ_p_ “2.2 (1.31,3.98)”, σ_t_ “0.13 (0.09,0.31)”] and ultimate elastic modulus (E_2_ “14.10 ± 7.21”) of adaptive intimal thickening were greater than those of other groups, the strain (ε_p_ “0.82 ± 0.17”, ε_t_ “0.53 ± 0.14”) of pathological intimal thickening was the largest in the pathological group.

**Conclusion:**

The present study systematically analyzed the influence of age, sex, test direction, anatomical site, and pathological characteristics on the biomechanical properties of the aorta, described the distribution of aortic atherosclerosis, and illustrated the characteristics of aortic thickness changes. At the same time, new insights into the grouping of pathological features were presented.

## 1 Introduction

The aorta, the largest blood vessel in the human body, is rich in multiple layers of elastic membranes and many elastic fibers (Sokolis, 2007). The anatomy of the aorta influences how well it functions. Atherosclerosis (AS) is the result of the aorta’s constant growth, aging, blood flow, or other diseases. These factors alter the aorta’s structure, particularly the breakdown or damage of elastic fibers and the proliferation of collagen fibers, which narrows the lumen and hardens the wall of the artery, and increases the risk of other cardiovascular diseases (aneurysm, arterial dissection, intramural hematoma, etc (Mussa et al., 2016; Herrington et al., 2018)). The death rate in forensic cases involving aortic rupture can reach 80%–94% (Ye et al., 2022). In particular, the co-occurrence of external violence and pathological tissue changes complicates cases of death from aortic rupture following traffic accidents or medical mishaps.

The biomechanical properties of the aorta are indispensable data for establishing a finite element model of the aorta (Garcia–Herrera et al., 2016) and performing computational hemodynamics based on fluid-structure interaction (Qiao et al., 2023). In addition, it provides important basic data in clinical surgical treatment and biomaterial development (Sherifova and Holzapfel, 2019). The current research is based on *in vitro* mechanical testing (for example, uniaxial tension (Franchini et al., 2021), biaxial tension (Pukaluk et al., 2022), peeling test (Myneni et al., 2020), bulge inflation tests (Romo et al., 2014), etc.) to evaluate the mechanical properties of the aorta. Uniaxial tensile testing is one of the most commonly used methods. Its advantages include loose test conditions, many types of test samples (for example,: brain tissue (Zwirner et al., 2020), lungs (Bel-Brunon et al., 2014), trachea (Teng et al., 2012), liver (Estermann et al., 2021), etc.), and relatively simple operation (Li et al., 2023). Some researchers prepared the aorta as a long strip for tensile testing (Kobielarz et al., 2020; Franchini et al., 2021), but Yi-Jiu Chen et al. confirmed that dog bone-shaped samples are better suited for tensile testing (Pei et al., 2021). Numerous academics have examined the aorta’s mechanical properties from a variety of perspectives, including age (Haskett et al., 2010), gender (Sokolis et al., 2017), pathological features (Bai et al., 2018), different segments (Peña et al., 2019), and test directions (axial and circumferential) (Franchini et al., 2021; Polzer et al., 2021), and so forth.

Some studies summarised the variability in previous test results (Walsh et al., 2014; Sherifova and Holzapfel, 2019), and a lack of systematic research due to the absence of uniform test standards in earlier studies (such as the geometric shape of the sample, etc.). In addition, Fewer studies have provided a more detailed description of the mechanical properties of the aorta in middle-aged and elderly people. At the same time, we provide new insights into the pathological grouping of the aorta, which may provide some reference value for subsequent studies.

Based on the above issues. The current study used a fresh aorta removed during autopsy. Basic data, such as age and sex, was then gathered. The aorta was first observed grossly morphologically. Next, samples of the aorta were taken in two directions (circumferential and axial) from four anatomical regions: the ascending aorta, aortic arch, thoracic aorta, and abdominal aorta. Then, a thickness measurement was taken on a dog bone-type sample, and a uniaxial tensile test was conducted. Histological staining was used to classify the aorta into four groups: Normal, Adaptive intimal thickening, Pathological Intimal thickening, and Fibrous atherosclerosis group. Finally, statistical analysis was carried out.

## 2 Materials and methods

### 2.1 Specimen collection

This study was approved by the Ethics Committee of Chongqing Medical University and informed consent was given by the deceased’s next of kin.

The aorta (death within 24 h, no aortic disease, and intact aortic structure) and pertinent information were obtained from the autopsy of the Forensic Medicine Department of Chongqing Medical University (Chongqing Forensic Injury Examination Institute). The entire aorta was collected and transferred to the laboratory in a crisper box with phosphate buffer at an internal temperature of 4°C–5°C.

### 2.2 Uniaxial tensile test

#### 2.2.1 Tensile test configuration

**FIGURE 1:**
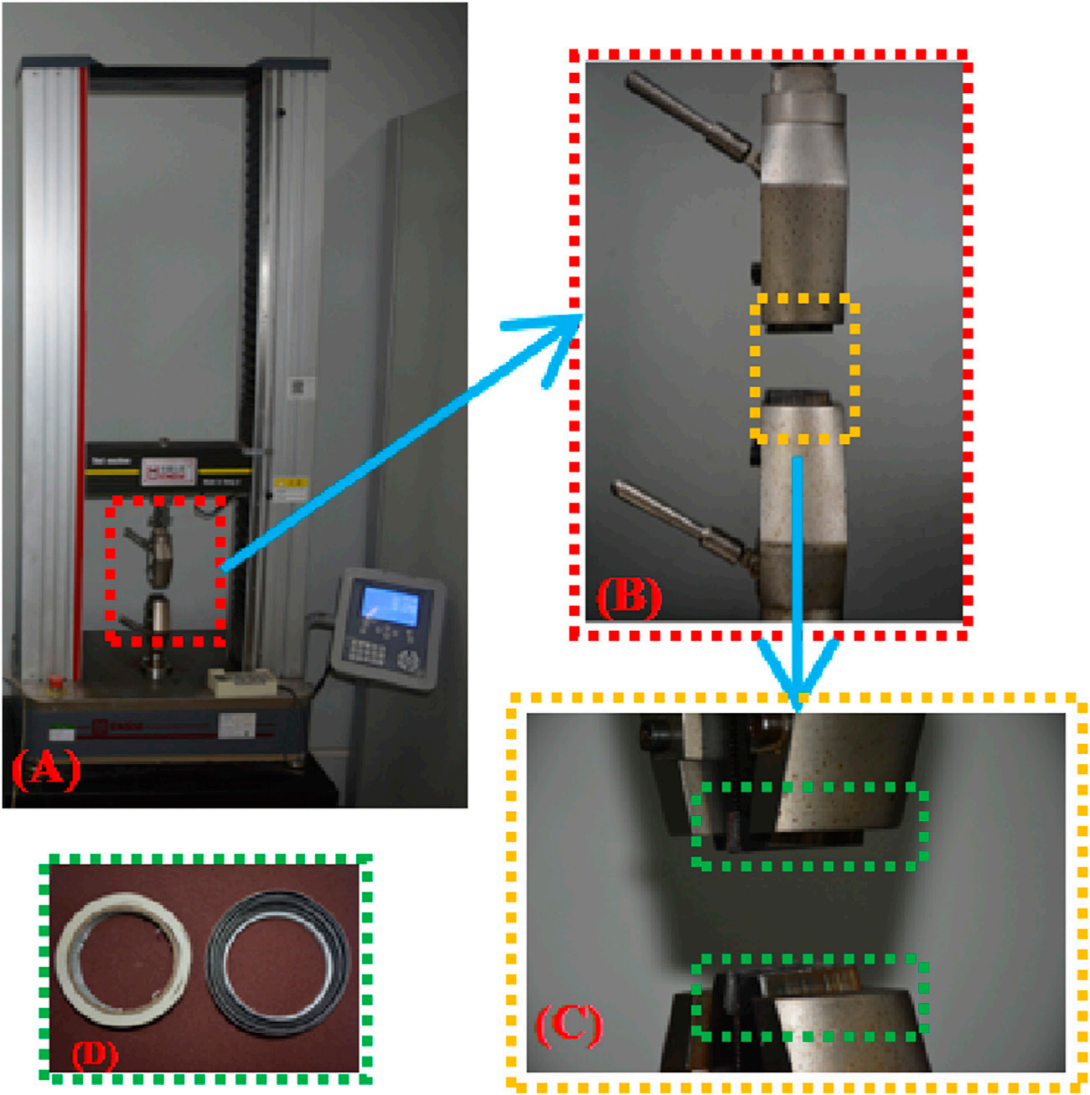
**(A)** Axial tensile test platform; **(B)** Clamp; **(C)** Buffer strip added inside the clamp; **(D)** Buffer strip material.

The tensile test was carried out using an electronic universal material testing machine that was controlled by a microcomputer (Figure 1). A buffer strip with a frosted paper strip was adhered to the inside of the clamp to lessen the force the clamp applied to the sample and its tendency to slip. In operation, the upper clamp stretches until the sample breaks at a quasi-static speed of 4 mm/min after going through three preconditioning cycles (about 5% stretching at a speed of 4 mm/min) to get rid of the hysteresis effect.

**FIGURE 2:**
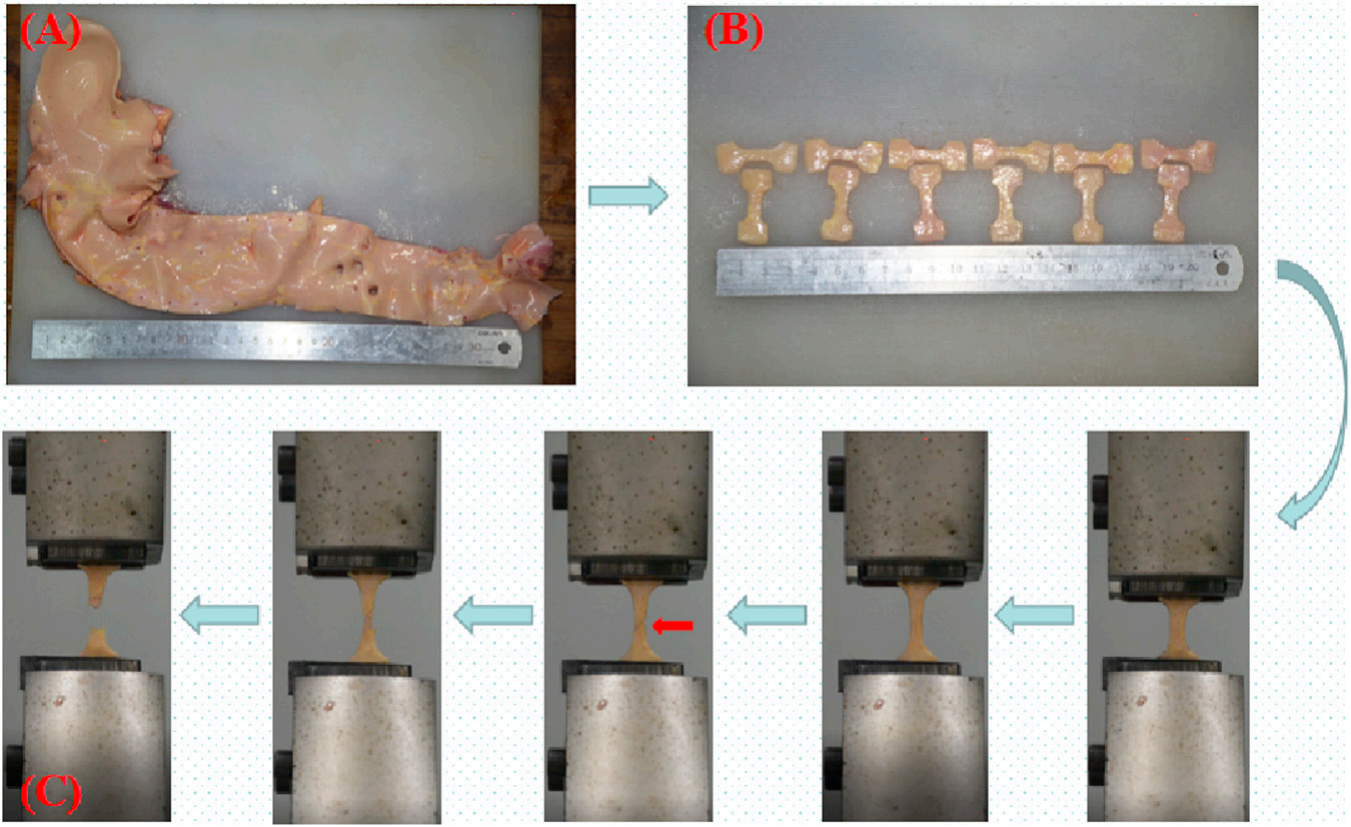
**(A)** Complete aorta; **(B)** Dog bone-shaped aorta sample; **(C)** Uniaxial tensile test process; the red arrow indicates the beginning of intimal rupture.

Before the test, the adventitia’s surrounding connective tissue was cut away, and aortic branch vessel openings were avoided, 174 dog-bone-shaped samples (where “N" denotes the number of samples) were created using a stamping die. Use a vernier caliper (accurate to 0.01 mm) to measure the original thickness (measure three times, taking the average value), the clamp clamped both ends of the sample and started running. The fracture near the middle of the sample was judged as a valid sample (Figure 2C) (Loree et al., 1994; Pei et al., 2021), there were 156 valid samples (accounting for 89.66%). The number of samples in each group is shown in Table 1. The test was completed within 48 h (Walsh et al., 2014).

**TABLE 1:** Number of samples in each group.

Factor	Group	N
Age	Range (54‐88 years)	156
Gender	Male	98
	Female	58
Anatomic Location	Ascending Aorta	21
	Aortic Arch	40
	Thoracic Aorta	54
	Abdominal Aorta	41
Direction	Circumferential	80
	Longitudinal	76
Anatomic Location	Normal	32
	Adaptive Intimal Thickening	16
	Pathological Intimal Thickening	68
	Fibrous Atherosclerosis	40

#### 2.2.2 Test data processing

**FIGURE 3:**
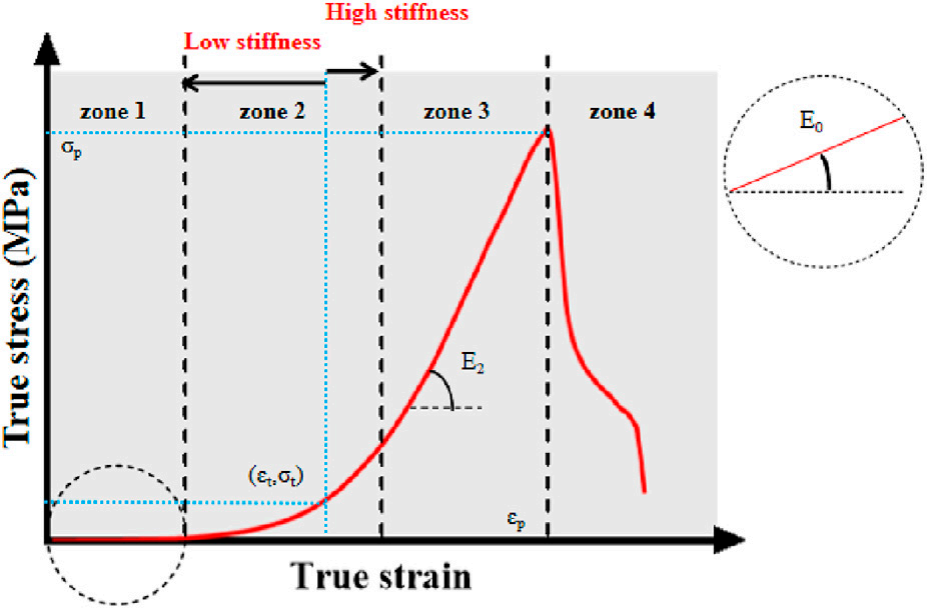
True stress-strain curve. Note: σ_p_-ultimate stress; ε_p_-ultimate strain; E_2_-ultimate modulus of elasticity; σ_t_-middle stress point; ε_t_-middle strain point; E_0_-starting modulus of elasticity. Zone 1, 2, 3, and four denote the starting, transition, strengthening, and failure phases of the aortic tensile test, respectively.

We depict the real stress-strain because it more accurately captures the dynamic mechanical properties of the sample in real time. The stress, strain, and elastic modulus at each stage can be obtained using a curve graph (Figure 3). We depict the real stress-strain because it more accurately captures the dynamic mechanical properties of the sample.

The raw data (load F, displacement L1 ) are processed to obtain the true stress and strain.

The specific processing is as follows:

Assuming that the aortic tissue is incompressible (Chuong and Fung, 1983; Wang et al., 2023), the sample initial thickness T0, width W0 , length L0 , the sample thickness T , width W , length L during stretching, then:

L=L0+L1 (1)

W0 ·T0·L0=W·T·L (2)

The initial cross-sectional area A0 and the current cross-sectional area A , then:

A0·L0=A·L (3)

A0=W0·T0 (4)

$$A=\frac{A0\cdot L0}{L}$$ (5)

The true stress is the ratio of the current load to the current cross-sectional area and is mathematically formulated as:

$$\sigma T=\frac{F}{A}=\frac{FL}{A0\cdot L0}$$ (6)

The true strain is the natural logarithm of the ratio of the current length to the initial length, and its mathematical formula is:

$$\varepsilon T=\ln\left(\frac{L}{L0}\right)$$ (7)

The modulus of elasticity is the stress required for the elastic deformation of a material produced by an external force. It is an indicator of the material’s ability to resist elastic deformation, usually expressed as E . The modulus of elasticity is a measure of the material’s ability to resist elastic deformation. The bigger the value, the more rigid the material is and the more stress it experiences during a certain elastic deformation. Defined as the ratio of stress to strain in the elastic deformation stage of a material:

$$E=\frac{d\sigma T}{d\varepsilon T}$$ (8)

The graph has four stages. In the first, alterations in elastic fibers predominate when subjected to physiological stresses. During the transition stage, the slope gradually increases as collagen fibers engage in the activity, indicating hyperelasticity (Li et al., 2023; Wang et al., 2023); during the reinforcing stage, the slope reaches its maximum and the collagen fibers provide their greatest effect; during the failure stage, the sample completely shreds as the ultimate load is met (Utrera et al., 2022).

The middle variables (σ_t_, ε_t_) are obtained based on the first order derivatives of the ture stress-strain curves ( dσT/dεT ). In this curve, the midpoint of the transition zone is defined in this study as the middle variable (García-Herrera et al., 2012).

### 2.3 Histological staining and classification

After testing, the samples were put in an embedding box and fixed for 24 h with 10% formalin added. After paraffin slices (4 μm in thickness) were made, they were stained with HE (hematoxylin and epoxy resin), which showed red coloration of the cytoplasm as well as the extracellular matrix, and bluish-purple coloration of the chromatin in the nucleus and nucleic acids in the cytoplasm as well as the calcium deposit under a light microscope.

**FIGURE 4:**
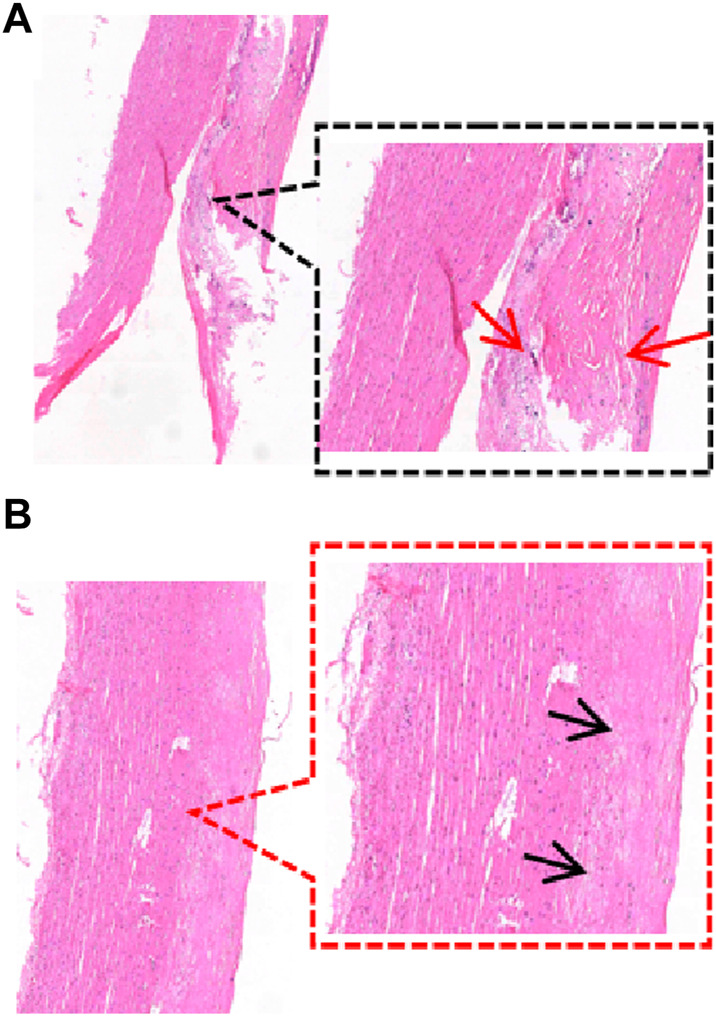
Different regions of a sample. **(A)** The sample’s broken end exhibits thickening of the intimal collagen fibers, calcium salt deposits, cholesterol crystals under the fibrous cap (HE staining reveals needle cracks), and foam cell formation within the necrotic core (red arrows), defined as fibrillary atherosclerosis; **(B)** The sample’s mid-section exhibits thickening of the intima of, with the presence of acellular lipid pools in the intima, and near the media where lipid deposition and proteoglycans are abundant but SMCs are absent (black arrows), defined as pathological intimal thickening. (HE staining, 40×, 80×).

**FIGURE 5:**
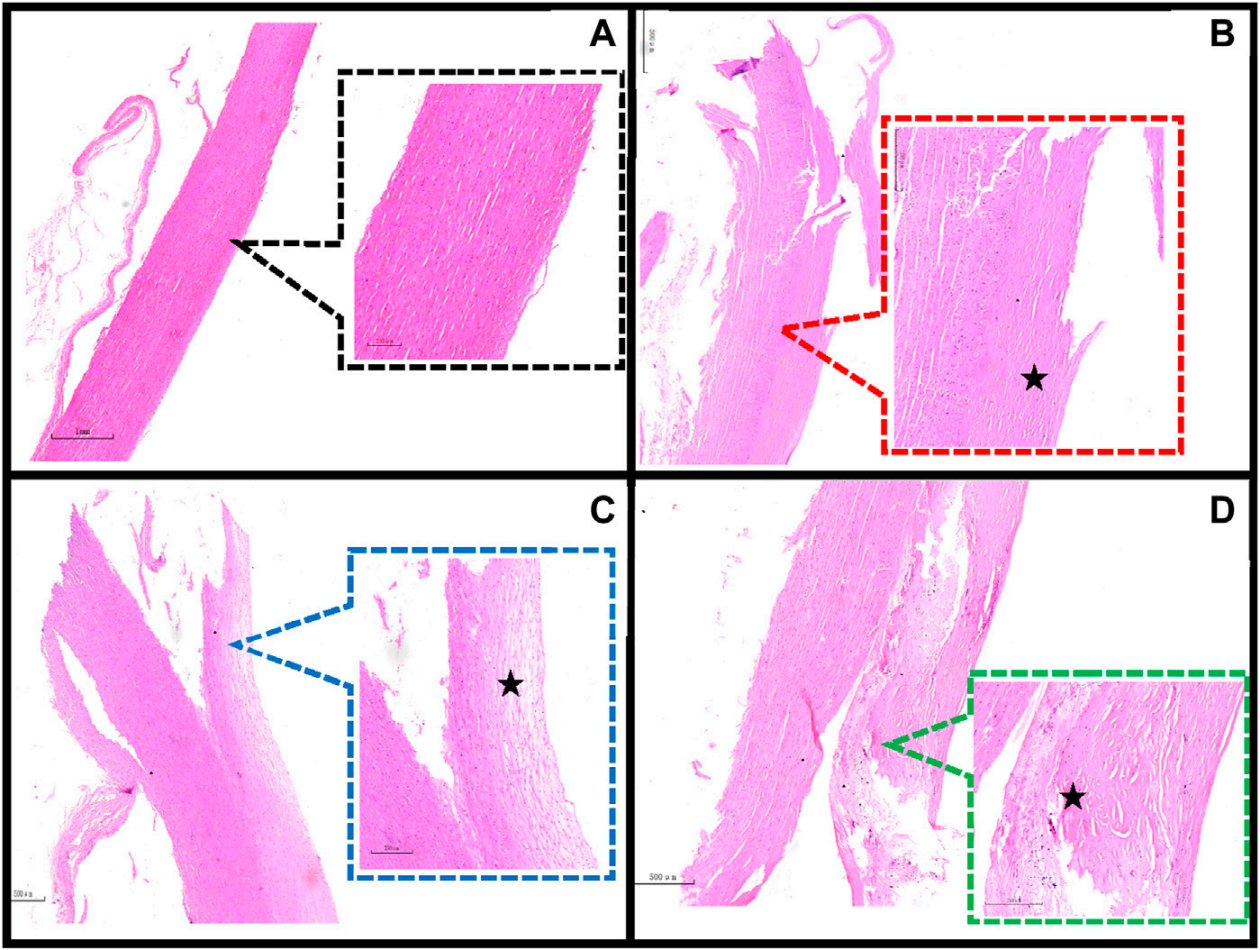
Classification of the main pathological features in the region of the sample dissection. **(A)** Normal aorta (20×, 80×); **(B)** Adaptive intimal thickening is characterized by intimal thickening begins with an increase in smooth muscle cells (SMCs) and proteoglycan-collagen matrix with little or no infiltration of inflammatory cells (40×, 80×); **(C)** Pathological intimal thickening is characterized by the intima has many fusiform, foamy cells clustered beneath the arterial endothelium, and in some areas of the intima there are numerous proteoglycan and lipid deposition of lipid pools (80×, 200×); **(D)** Fibrillary atherosclerotic is characterized by calcium salt deposits and cholesterol crystals under the fibrous cap (HE staining shows needle cracks), foam cell formation and infiltration of inflammatory cells are seen within the necrotic core (40×, 80×). ★ indicates the main lesion area. Additional note: Figure 4 and Figure 5D are taken from the same sample.

Previous studies have taken tissue from neighboring samples for histological staining (Kobielarz et al., 2020; Pukaluk et al., 2022), whereas there may be different pathological features between the sample and the neighboring area or in different areas of the same sample (Figure 4). Therefore, we observed the pathological features of the samples by taking the broken end of the sample as the weakest point and then grouped them according to the development of atherosclerosis (normal group, adaptive intimal thickening group, pathological intimal thickening group, fibrous atherosclerosis group) (Otsuka et al., 2014; Jing et al., 2022) (Figure 5).

## 3 Statistical analysis

SPSS 24.0 software was used for statistical analysis and Graphpad Prim9 for plotting. Mean ± standard deviation (Mean ± SD) was used for normally distributed measures, and median (interquartile range) [M(P25, P75)] was used for non-normally distributed measures; t-test or Mann-Whitney U-test was used for comparisons between two groups; one-way ANOVA or Kruskal-Wallis test was used for comparisons between three or more groups; Indicators with significant differences were tested by LSD multiple comparisons or Kruskal-Wallis pairwise comparison test; regression analysis was carried out by linear regression method; when a *p*-value was less than 0.05, it was assumed to be significant, and was denoted by "*", "**" indicates *p* ≤ 0.01, and "***" indicates *p* ≤ 0.001.

## 4 Results

### 4.1 Gross morphological observations

**FIGURE 6:**
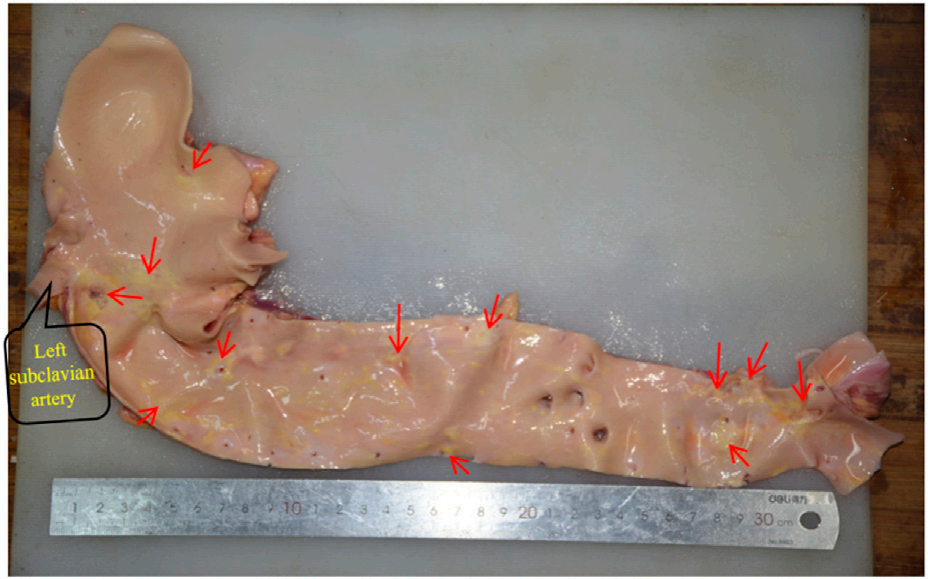
The distribution of aortic atherosclerosis. The concentrated region of hardened plaques is indicated by the red arrow.

**FIGURE 7:**
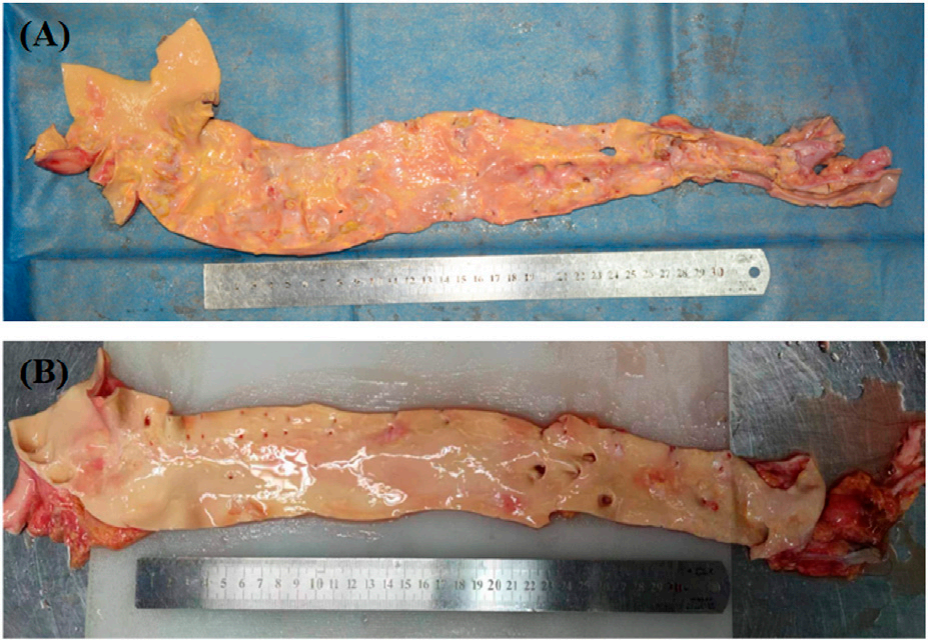
**(A)** Male, 58 years old; **(B)** Female, 58 years old.

The pathological alterations in the aortic intima are shown in Figure 6. We found that the distribution of the segments was most severe in the abdominal aorta, followed by the aortic arch, and least severe in the ascending aorta. Atherosclerotic plaques were mostly seen in the openings of the vascular branches, particularly at the left subclavian artery opening and at the branches of the common iliac artery. When comparing the two genders, males had a more severe tendency of aortic atherosclerosis (Figure 7).

### 4.2 Comparison of thickness

**FIGURE 8:**
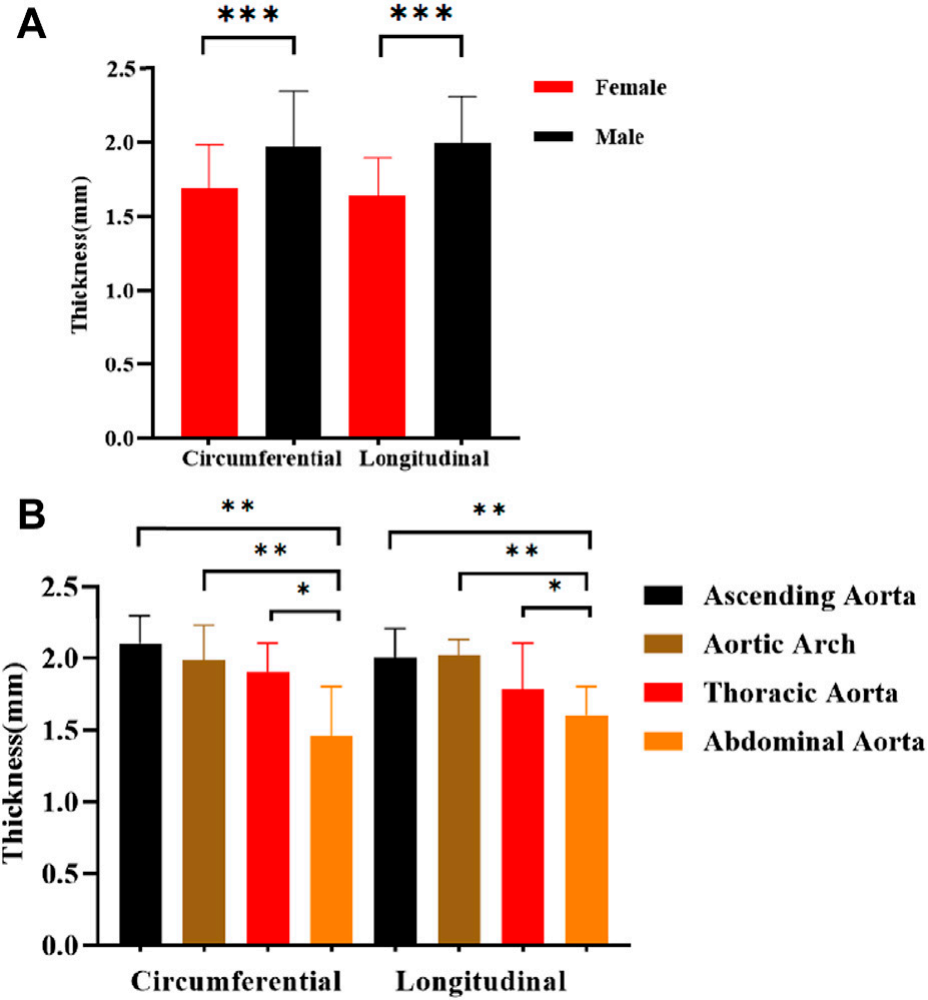
Comparison of thickness in different directions of stretching. **(A)** Different genders; **(B)** Different anatomical parts.

Between age and thickness, there is no discernible linear connection. Comparisons of thicknesses between anatomical locations, test orientations, and genders are shown in Figure 8. The axial and circumferential directions do not significantly differ from one another. Males’ average thickness was noticeably higher than females’ when it came to gender. The abdominal aorta is substantially thinner than the aortas in other anatomical regions, and the aorta thickness gradually declines from the proximal to the distal end.

### 4.3 The relationship between age and parameters

**FIGURE 9:**
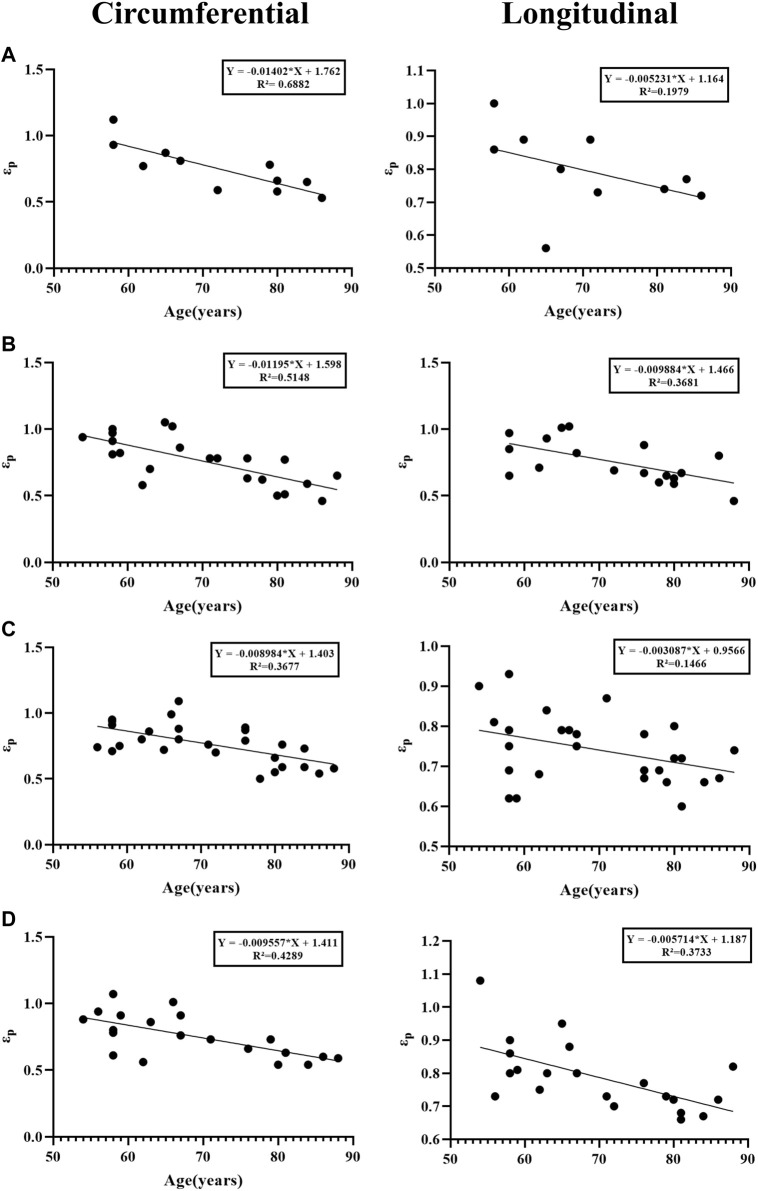
Linear regression analysis between age and strain. **(A)** Ascending aorta; **(B)** Aortic arch; **(C)** Thoracic aorta; **(D)** Abdominal aorta.

There is a negative correlation between age and several parameters in different anatomical regions. The most significant one is strain (ε_p_, ε_t_), which means that as age increases, strain gradually decreases (Figure 9), especially in the circumferential ascending aorta (ε_p_ “Y = −0.01402*X + 1.762, R^2^ = 0.6882”, ε_t_ “Y = −0.01062*X + 1.250, R^2^ = 0.6772”). In the axial direction of the ascending aorta, age is related to stress (σ_p_ “Y = −0.02607*X + 3.018, R^2^ = 0.3557”, σ_t_ “Y = −0.005977*X + 0.8938, R^2^ = 0.5066”) and elastic modulus (E_2_ “Y = −0.1346*X + 14.82, R^2^ = 0.5125”, E_0_ “Y = −0.001010*X + 0.1091, R^2^ = 0.4545”).

### 4.4 Comparison of parameters for different genders

**FIGURE 10:**
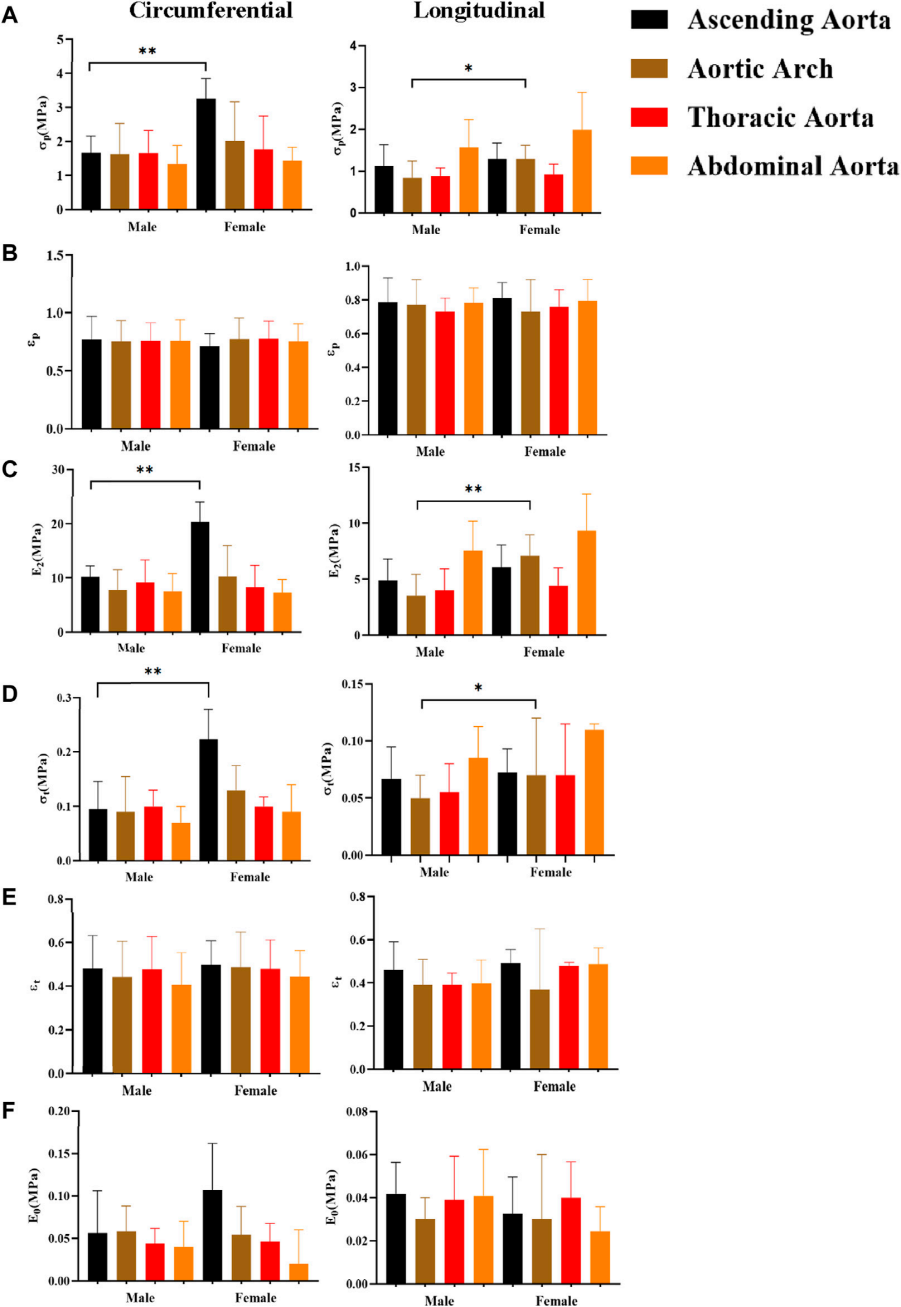
Comparing the effect of gender on mechanical parameters. **(A)**, **(B)**, **(C)**, **(D)**, **(E)**, and **(F)** denote the ultimate stress (σ_p_), ultimate strain (ε_p_), ultimate modulus of elasticity (E_2)_, middle stress point (σ_t_), middle strain point (ε_t_), and initial modulus of elasticity (E_0_), respectively.

The influence of gender on mechanical characteristics in various orientations at various anatomical regions is shown in Figure 10. In general, females exhibit more dominance, as evidenced by statistically significant differences in stress (σ_p_, σ_t_) and ultimate modulus of elasticity (E_2_). The gender differences were more significant in the circumferential direction of the ascending aorta (σ_p_ “3.25 ± 0.59”, σ_t_ “0.23 ± 0.11”, E_2_ “20.28 ± 3.74”), and in the axial direction of the aortic arch [σ_p_ “1.28 ± 0.33”, σ_t_ “0.07 (0.06,0.12)”, E_2_ “7.09 ± 1.87”]. In addition, we separated into normal and atherosclerotic groups to compare the parameters between genders (Table 2). In the normal group, the parameters were greater in males, ultimate stress [σ_p_ “1.20 (1.00,1.85)”] and ultimate strain (ε_p_ “0.76 ± 0.13”) were the most significant differences, while in the atherosclerotic group, the parameters were greater in females, and all parameters have significant differences [except initial modulus of elasticity (E_0_) and ultimate strain (ε_p_)].

**TABLE 2:** Comparison of parameters between genders in normal aorta and atherosclerotic groups.

Sex	Pathology	σ_p_	ε_p_	E_2_	σ_t_	ε_t_	E_0_	N
Male	Normal	1.20 (1.00,1.85)^*^	0.76 ± 0.13^*^	7.45 ± 3.39	0.07 (0.05,0.11)	0.43 ± 0.10	0.05 (0.03,0.07)	19
	Atherosclerosis	1.24 (0.78,1.75)^**^	0.76 ± 0.15	6.88 (3.76,9.16)^**^	0.07 (0.05,0.10)^***^	0.42 (0.32,0.49)^**^	0.06 (0.04,0.07)	79
Female	Normal	0.95 (0.71,1.34)^**^	0.66 ± 0.11^*^	6.27 ± 2.78	0.06 (0.05,0.09)	0.37 ± 0.08	0.03 (0.02,0.05)	12
	Atherosclerosis	1.58 (1.21,2.23)^*^	0.79 ± 0.13	7.77 (5.67,11.14)^**^	0.10 (0.07,0.14)^***^	0.48 (0.40,0.57)^**^	0.04 (0.02,0.06)	46

### 4.5 Comparison of parameters in different test directions

**FIGURE 11:**
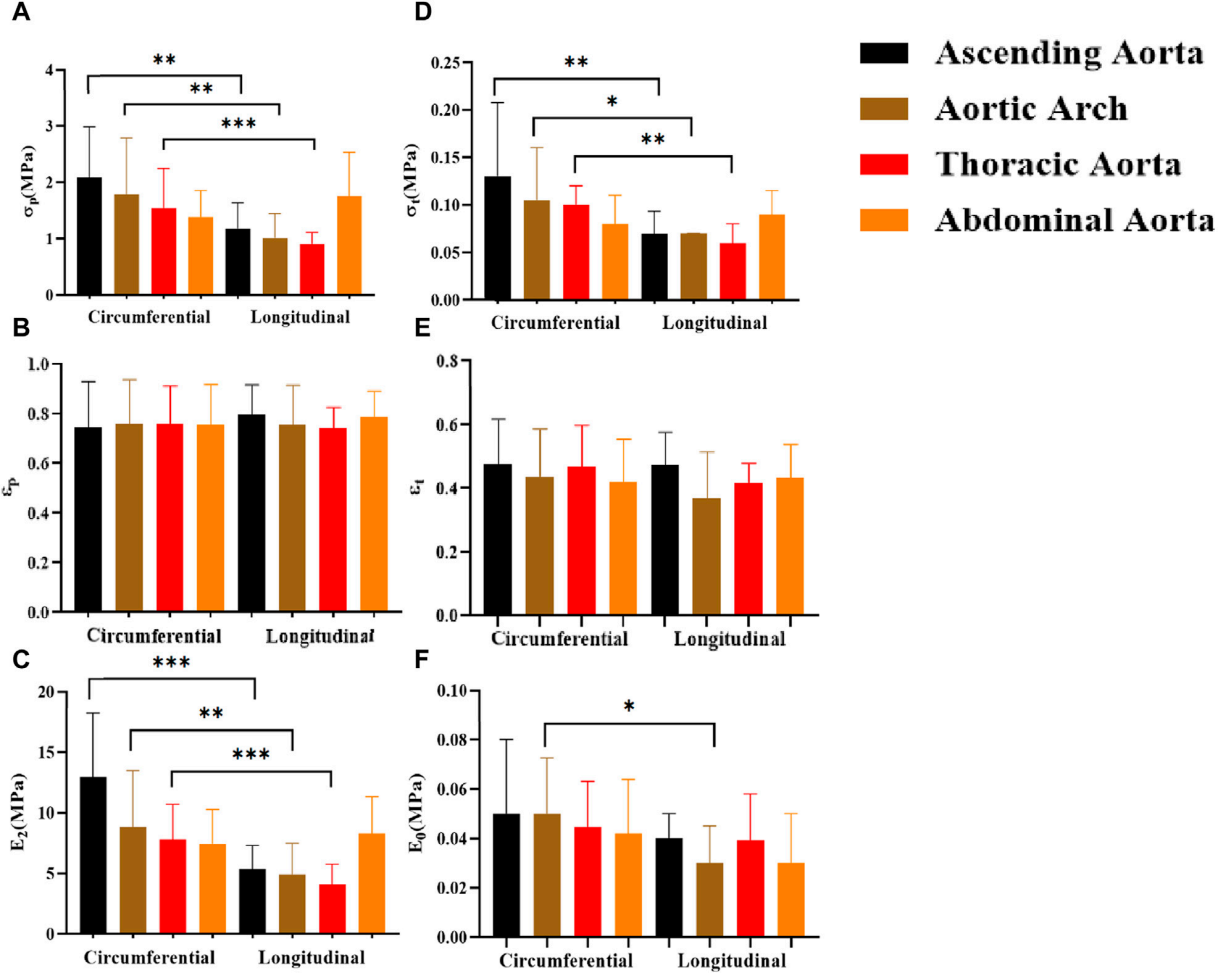
Comparison of the effects of different test orientations on mechanical parameters. **(A–F)** denote the ultimate stress (σ_p_), ultimate strain (ε_p_), ultimate elastic modulus (E_2_), middle stress point (σ_t_), middle strain point (ε_t_), and initial elastic modulus (E_0_), respectively.

In general, the mechanical characteristics exhibit higher values in the circumferential direction as compared to the axial direction (Figure 11). Except for the abdominal aorta group, there were significant differences in stress (σ_p_, σ_t_) and ultimate modulus of elasticity (E_2_), whereas the initial modulus of elasticity (E_0_) showed significant differences only in the aortic arch.

### 4.6 Comparison of parameters at different anatomical parts

**FIGURE 12:**
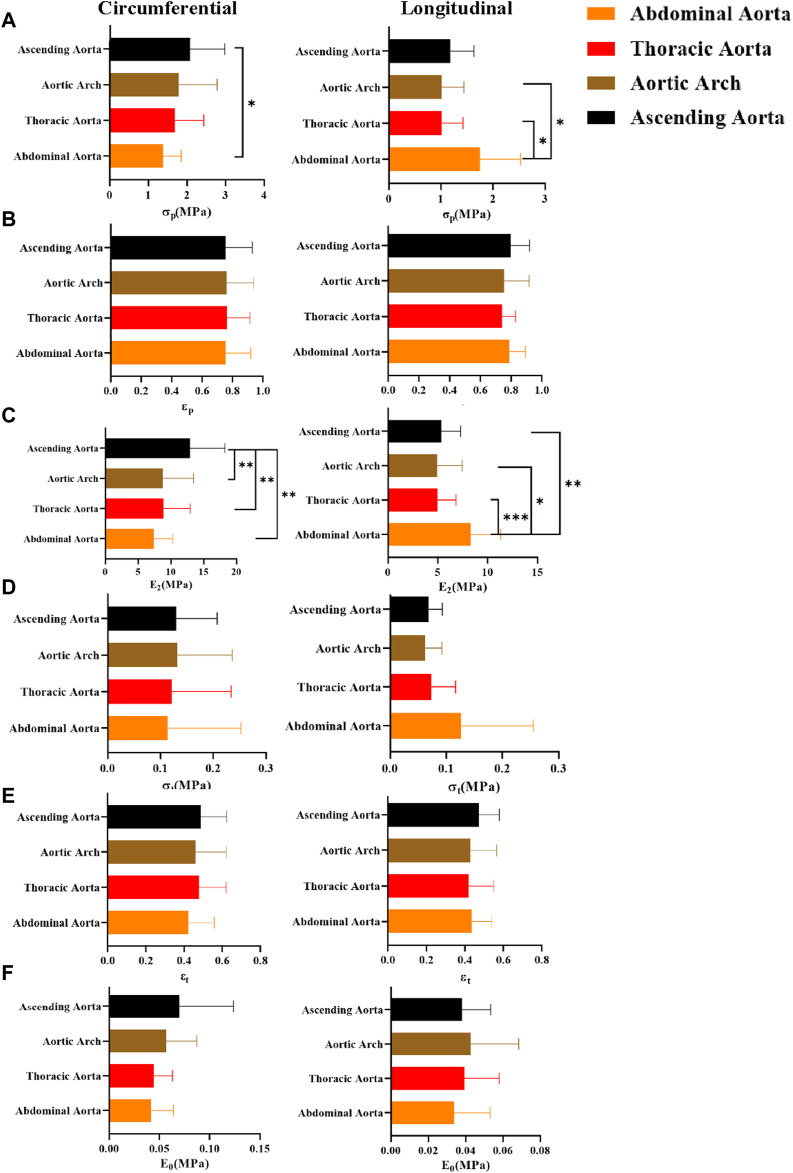
Comparison of mechanical parameters at different anatomical parts. **(A–F)** denote the ultimate stress (σ_p_), ultimate strain (ε_p_), ultimate modulus of elasticity (E_2_), middle stress point (σt), middle strain point (ε_t_), and initial modulus of elasticity (E_0_), respectively.

The mechanical parameters of the ascending aorta were greater in the circumferential direction, with the most significant higher ultimate stress (σ_p_ “2.09 ± 0.89”) and ultimate modulus of elasticity (E_2_ “12.93 ± 5.29”). On the other hand, the abdominal aorta had significantly greater ultimate stress [σ_p_ “1.69 (1.08,2.32)”] and ultimate modulus of elasticity [E_2_ “8.28 (6.67,10.25)”] than the rest of the part in the axial direction (Figure 12). In the initial and transitional phases, the differences were not significant, but the ascending aorta still had a strong advantage.

### 4.7 Comparison of parameters for different pathological features

**FIGURE 13:**
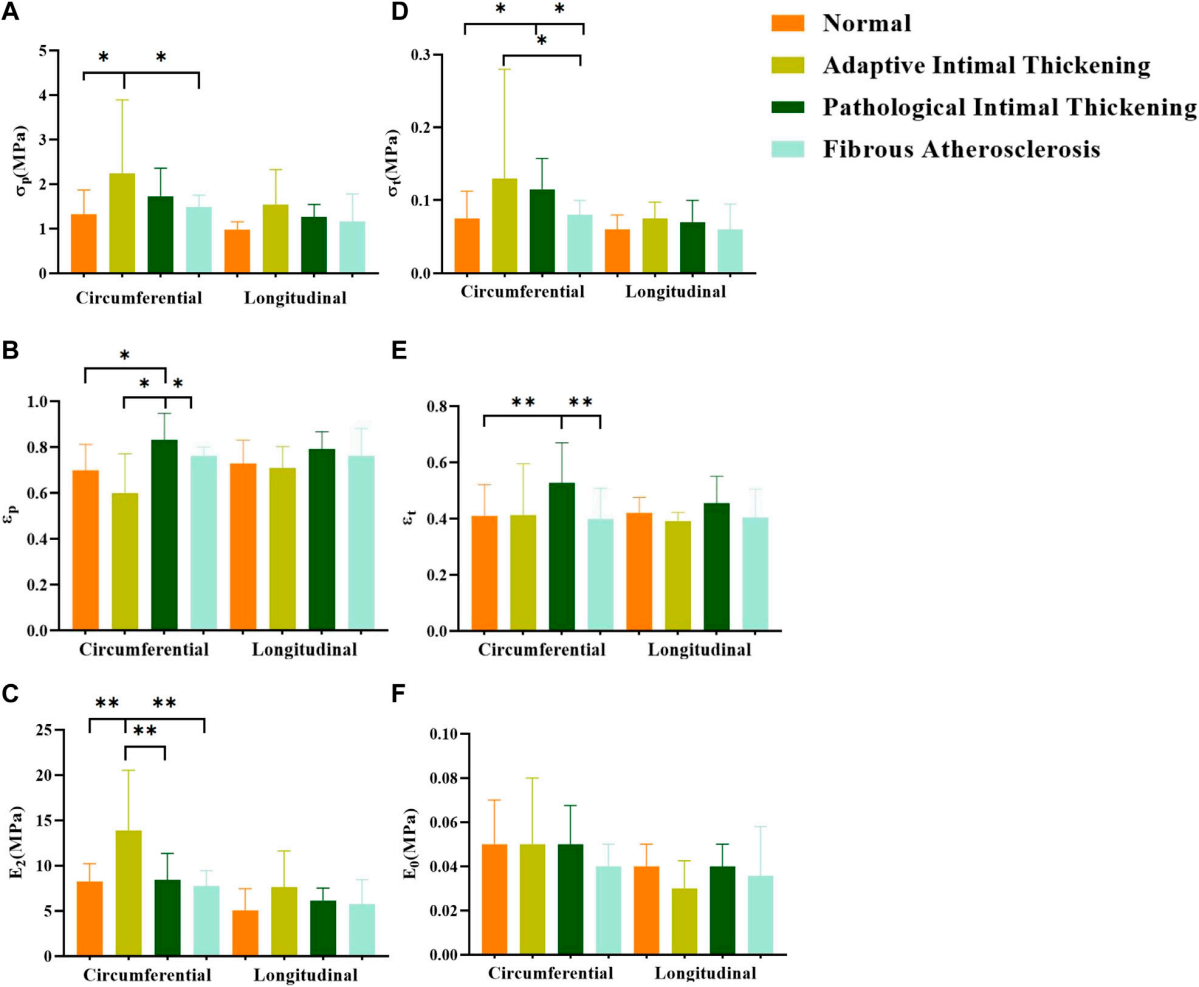
Comparison of parameters for different pathological features. **(A–F)** denote the ultimate stress (σ_p_), ultimate strain (ε_p_), ultimate modulus of elasticity (E_2_), middle stress point (σ_t_), middle strain point (ε_t)_, and initial modulus of elasticity (E_0_), respectively.

In general, the mechanical parameters tended to increase and then decrease with the development of atherosclerosis. In the circumferential direction, the stresses [σ_p_ “2.2 (1.31,3.98)”, σ_t_ “0.13 (0.09,0.31)”] and ultimate modulus of elasticity (E_2_ “14.10 ± 7.21”) of the adaptive Intimal thickening group were significantly greater than the other groups, whereas the strain (ε_p_ “0.82 ± 0.17”, ε_t_ “0.53 ± 0.14”) was greater in the pathological Intimal thickening group. There was no statistically significant difference in the parameters of the axial direction (Figure 13).

## 5 Discussion

This study systematically analyzed the mechanical parameters of the aorta under the influence of age, gender, test direction, anatomical location, and pathological characteristics. In addition, we elaborated on the distribution of aortic atherosclerosis through gross morphological observation. The characteristics of aortic thickness changes were analyzed. At present, there is no relatively comprehensive study.

### 5.1 Comparison of thickness and atherosclerosis distribution

There are certain differences in aortic thickness with age, gender, and anatomical location (Albu et al., 2022), which is attributed to its composition on the one hand and atherosclerosis and intimal thickening on the other hand (Li et al., 2004). In the present study, there is no discernible trend in aortic thickness at 50 years of age and above. When comparing genders, the Male group had greater thickness (Li et al., 2004) and higher prevalence and extent of atherosclerosis than the female group (Mathur et al., 2015), the reasons could be increased endothelial fibroblast proliferation and accumulation of collagen fibers, and muscle sympathetic nerve activity (MSNA) was greater in males, stimulating smooth muscle hypertrophy, which was not present in females (Holwerda et al., 2019). In terms of anatomical parts, the thickness of the aorta decreases progressively from proximal to distal, which is mainly related to the structural composition of the aorta, where elastin, collagen, and smooth muscle form the laminar units, which are the structural and functional units of the aorta (O Connell et al., 2008). Arteries in the proximal part of the aorta have denser laminar units.

Among the segments, the abdominal aorta has the most severe atherosclerosis, followed by the aortic arch, and the ascending aorta is the least severe. The abdominal aorta has fewer laminar units, poor elasticity, and is itself a high-incidence part of endothelial injury (Czamara et al., 2020). In addition, the abdominal aorta has more macrovascular branches (abdominal celiac trunk arteries and common iliac arteries, among others), which makes it more susceptible to vortex phenomena (Qin et al., 2021). The unique geometric features of the aortic arch make it subject to higher shear forces, especially at the opening of the left subclavian artery.

### 5.2 The effect of age on parameters

Despite the age limitation of the present study, the results show parameters gradually decrease with age, which is consistent with previous studies (Huh et al., 2019). The most significant difference of these was strain (ε_p_, ε_t_). It could be due to weaker or reduced fiber cross-linking caused by structural elastin and collagen degeneration (Astrand et al., 2011), decreased density of elastin and smooth muscle (Jadidi et al., 2021), thicker intima-media collagen fibers, but no major change in overall collagen content. These factors remodel the aortic structure (Morrison et al., 2009; Albu et al., 2022), and have an impact on the aorta’s mechanical properties.

### 5.3 The effect of gender on parameters

In the healthy group, the mechanical parameters of males were greater (Ninomiya et al., 2015; Li et al., 2023), whereas, in the atherosclerotic group, the mechanical parameters of females were higher, especially in the proximal part of the aorta (ascending aorta and aortic arch) (Figure 8). The samples were taken from postmenopausal females. Estrogen mainly acts on endothelial cells and smooth muscle cells in premenopausal females, promoting the release of vasoactive mediators and mediating the functional balance of vascular inflammation, lipid metabolism, and oxidative stress (Zahreddine et al., 2021). For premenopausal females, the prevalence of atherosclerosis is lower than that of males (Man et al., 2020). After menopause, females’ ovarian function decreases, dyslipidemia increases (Peters et al., 1999), vascular smooth muscle cells migrate or proliferate, and oxidative stress and inflammatory reactions aggravate. Generally speaking, the prevalence of atherosclerosis increases in postmenopausal females, and the stiffness of the female vasculature increases (Zhang et al., 2022). Findings also suggest that the effects of estrogen may be concentrated in the proximal aortic region (Waddell et al., 2001).

### 5.4 The effect of different test directions on parameters

The circumferential direction is the primary orientation of the aortic laminar unit (O Connell et al., 2008; Holzapfel and Ogden, 2018), and the mechanical characteristics of the aorta exhibit anisotropy (Sigaeva et al., 2019; Pukaluk et al., 2022). The result showed that the parameters of the circumferential direction were greater than the axial direction group, in line with previous studies (Gaur et al., 2020), but there were no significant differences in the abdominal aorta. It may be due to an increased gradually proportion of collagen fibers from proximal to distal, which are predominantly aligned in a longitudinal direction (Assoul et al., 2008). Meanwhile, the ascending aorta is subjected to the greatest blood pressure and wall shear, whereas the descending aorta is subjected to less blood pressure and wall shear based on the results of computational hemodynamic analysis (Malvindi et al., 2017). This may explain the decreasing prevalence of aortic dissection from the ascending aorta to the distal end of the abdominal aorta, and the fact that the tear direction of dissection tends to be transverse (Sherk et al., 2021). The structural and hemodynamic characteristics of the abdominal aorta may be made intima less prone to tear, and then form isolated abdominal aortic dissection (Sen et al., 2021), but it more prone to abdominal aorta aneurysms (Song et al., 2023).

### 5.5 The effect of different anatomical parts on parameters

Currently, there are no studies of uniaxial stretching tests on four anatomical parts together. In the previous study, Ninomiya et al. found that the strength and elasticity of the thoracic aorta were higher than that of the abdominal aorta in circumferential stretching (Ninomiya et al., 2015). Our study showed that the ascending aorta had the greatest parameters in circumferential stretching, with the most significant differences in ultimate stress (σ_p_) and ultimate modulus of elasticity (E_2_). the ascending aorta has more laminar units, greater total fibronectin content and density, and a more complete structure (Emmott et al., 2016). In axial tension, the abdominal aorta had the greatest ultimate stress (σ_p_) and ultimate modulus of elasticity (E_2_) (Figure 10). As mentioned earlier, collagen fibers grow longitudinally and the abdominal aorta has a higher collagen fiber content, collagen fibers are stretched during the strengthening phase and elevate the abdominal aorta’s tensile strength.

### 5.6 The effect of different pathological features on parameters

With the development of aortic atherosclerosis, the mechanical parameters showed a tendency to increase and then decrease (Koniari et al., 2011). The adaptive intimal thickening group exhibited the highest stress (σ_p_, σ_t_) and ultimate modulus of elasticity (E_2_). This may be explained by the increased collagen fiber content and density in the intima, as well as the absence of pathological alterations including aberrant fiber structural organization and fibrin denaturation (Glagov et al., 1993; Herity et al., 1999). The strain (ε_p_, ε_t_) was significantly higher in the pathological intimal thickening group (multilayered foam cells or lipid pools with loose intima) than that of other groups, which suggested that the aorta stretches longer. The abnormal proliferation and migration of smooth muscle cells or macrophage infiltration gradually forms layers of lipid cells and foam cells (Bennett et al., 2016), resulting in the relaxation of the connective action between fibrous tissues. Compared with the fibrous atherosclerosis group, the normal group did not show significant differences in any of the parameters, but the modulus of elasticity (E_2_, E_0_) was slightly higher than that of the fibrous atherosclerosis group. Fibrous atherosclerosis is mainly due to the formation of lipid cores, atherosclerotic substances, or calcium salt deposits under the intima, resulting in a severe effect on the structure of the tunica intima and media tunica (Jing et al., 2022). To some extent, thickening of the intimal collagen fibers improves the aortic stiffness, but the internal structural integrity is damaged, and some of the elastic and collagen fibers are structurally and functionally impaired (Tsamis et al., 2013). A balance between damage and repair is achieved (Uimonen, 2021) to keep the original mechanical properties.

### 5.7 Outlook

Only human aorta samples older than 50 years old were collected for this research. In addition, the aorta is anisotropy, and multi-axial tensile testing may better simulate physiological conditions. Bulge inflation tests are a more demanding test condition method for multiaxial testing to determine ultimate properties (Duprey et al., 2016). The present study focuses on how different variables affect the aorta’s mechanical characteristics at different phases. In previous studies, some scholars have used optical extensometers to solve the strain problem, and our study uses the crosshead displacement to derive the strains, which may hinder the comparison with the results of previous studies but does not influence the study of the changing pattern of the mechanical properties of the aorta. In further research, we will make efforts to further illustrate the mechanical properties of the aorta by collecting samples (normal aorta, aortic dissection, aneurysm, etc.) in a targeted manner and selecting the optimal test method according to the required parameters.

## 6 Conclusion

This study concluded that the abdominal aorta is most susceptible to atherosclerosis. With the development of atherosclerosis, mechanical parameters decrease, and the risk of injury increases. The average thickness of the aorta was greater in males than that of females and decreased progressively from the ascending aorta to the abdominal aorta. Mechanical parameters were smaller in the axial direction, which may explain the greater risk of injury to blood vessels in axial stretching. For the grouping of pathological features, we proposed the pathological features of the sample’s broken end as the condition for grouping. In addition, we consider that stiffness may be greater in middle-aged and elderly females after the occurrence of atherosclerosis.

## Data Availability

The original contributions presented in the study are included in the article/Supplementary material, further inquiries can be directed to the corresponding author.
